# Cremation

**Published:** 1874-06

**Authors:** 


### ARTICLE VI.

### *Cremation.*

The subject of cremation is again taken up by Sir Henry Thompson in the pages of the *Contemporary Review.* In this article he replies to various criticisms that have appeared in different journals, and gives a detailed account of the process he would suggest as most appropriate for the object in view. Sir Henry states, and it is certainly a somewhat remarkable fact, that the only formal opposition to cremation has been made by the present medical Inspector of Burials for England and Wales, Mr. Holland; and in reply to the observations of this gentleman, Sir Henry refers to the evidence obtained by Drs. Southwood Smith, Waller, Lewis, and others, in regard to the large amount of gases produced in the decomposition of the body, and the impregnation of soil, water, and air to a considerable distance. Such impregnation by the dead, and consequent danger to the living, cannot, we presume, be questioned for a moment, and is fully borne out by the statements of Mr. Bowie, and the general experience of the profession. We must also fully endorse Sir Henry's remarks in regard to the elimination of ammonia, or at least of carbonate of ammonia, from decomposing animal tissues, and are at a loss to understand how any doubt can exist about the point. Turning to the second part of Sir Henry Thompson's essay, he remarks that he has personally superintended the burning of three bodies of animals, one weighing 47 lbs., another 140 lbs., and a third no less than 227 lbs., with the most satisfactory results, the residue in the first instance weighing only $1\frac{3}{4}$ lbs., and in the second 4 lbs. In the last case the body was placed in one of Dr. Siemen's furnaces, the interior of which was heated to about 2000° F. The inner surface of the cylinder, which was about 7 feet long by 5 or 6 feet in diameter, was smooth, almost polished, and no solid matter but that of the body was introduced into it. The gases, which were at first abundantly given off, passed through a highly heated chamber, among thousands of interstices made by intersecting fire-bricks laid throughout the entire chamber, lattice fashion, in order to minutely divide and delay the current, and to expose it to an immense area of heated surface. By this means they were rapidly oxidized, and not a particle of smoke issued by the chimney. No second furnace was therefore in this instance requisite, though, under certain circumstances, the products of combustion might be transmitted through another, and the fumes from this into a third, and so on, each being made available for the combustion of one body. The process was completed in fifty-five minutes, and the ashes, which weighed about 5 lbs., were removed with ease. Sir Henry meets the objection that has been raised to the practice of cremation, that it will lead to an increase of crimes of poisoning, by suggesting that a public verificator of the cause might be appointed, whose duty it should be to ascertain and certify the cause of death, whilst the stomach might be kept for some years. In reference to the expense, he still thinks it would be far within the present cost of a funeral. As regards ourselves, we have already expressed our opinion that it is an eminently satisfactory mode of disposing of the dead—safe, speedy, wholesome, and economical; but we rather doubt whether ancient custom and popular prejudice can be so easily overcome and altered as Sir Henry Thompson appears to believe.—*Lancet.*

---

### EDITORIAL, ETC.

